# Bedouin Children With Celiac Disease: Less Symptoms but More Severe Histological Features at Presentation

**DOI:** 10.3389/fped.2020.580240

**Published:** 2020-09-29

**Authors:** Baruch Yerushalmi, Sergei Vosko, Galina Ling, Ronit Raanan, Daniel L. Cohen, Haim Shirin, Tzipora Shalem, Shay Matalon, Efrat Broide

**Affiliations:** ^1^Pediatric Gastroenterology Unit, Soroka University Medical Center and the Faculty of Health Sciences, Ben-Gurion University, Beer-Sheva, Israel; ^2^The Gonczarowski Family Institute of Gastroenterology and Liver Disease, Shamir (Assaf Harofeh) Medical Center, Zerifin, Israel; ^3^The Jecheskiel Sigi Gonczarowski Pediatric Gastroenterology Unit, Shamir (Assaf Harofeh) Medical Center, Zerifin, Israel

**Keywords:** ethnicity, bedouins, consanguinity, Marsh criteria, celiac disease

## Abstract

**Background:** The prevalence of celiac disease (CD) has dramatically increased with wide variability in clinical presentations between different geographical areas. However, the contribution of ethnic disparities in pediatric celiac disease is still unclear, especially in patients of Bedouin origin.

**Objective:** We aimed to compare the clinical presentation and histological severity of celiac disease between Bedouin and Jewish children in southern Israel.

**Methods:** This is a retrospective study in which we collected the demographic and clinical data, laboratory results, and histological severity of CD in two ethnic groups: Bedouins and Jews. The study included patients who were diagnosed between 1997 and 2015 in a tertiary hospital in southern Israel.

**Results:** Data from 844 children with CD (271 Jewish and 573 Bedouins), 505 females (59.8%), were analyzed. Gastrointestinal symptoms and diabetes were more prevalent among the Jewish population (*p* < 0.001 and *p* = 0.008, respectively), while family history, failure to thrive, iron deficiency anemia, and histological severity were significantly more prevalent among the Bedouin group. Upon multivariate logistic regression analysis, only the presence of iron deficiency anemia and Bedouin origin were associated with more advanced histological disease (OR of 2.03 (95% C.I 1.31; 4.308) (*P* < 0.009) and OR 1.78 (95% C.I 1.31; 4.308) (*P* < 0.003) respectively).

**Conclusion:** The clinical presentation of celiac disease in Bedouin children is characterized by anemia with less gastrointestinal symptoms, but more severe histological damage. These differences might be explained either by a delay in the diagnosis of the disease in this population or by variable environmental, cultural, and nutritional factors unique to this ethnic group.

## Key Summary

### Summarize the Established Knowledge on This Subject

- Among different ethnic groups, the prevalence of celiac disease can vary widely
- The disease spectrum ranges from asymptomatic patients to patients with severe malabsorption symptoms

### What Are the Significant and/or New Findings of This Study?

- In the same region of Israel, two different populations (Jewish and Bedouin) present differently in terms of clinical characteristics and histological severity.
- Bedouin children presented more often with anemia, whereas Jewish children more frequently with gastrointestinal symptoms
- The histological severity of celiac disease was also more severe among Bedouins as compared to Jewish children

## Introduction

Celiac disease is an immune-mediated enteropathy triggered by the ingestion of gluten-containing grains in genetically susceptible individuals (1).

The prevalence of CD has increased dramatically over the last decade and it is considered to be one of the most common genetic disorders in Western countries with a prevalence of 1–2.67% (2–5). There is a wide range of clinical presentations of the disease at diagnosis. According to the ESPGHAN guidelines for the diagnosis of CD in children and adolescents, the symptoms and signs range from gastrointestinal symptoms and signs such as chronic diarrhea with malabsorption predominance, extra-intestinal symptoms and signs such as anemia, abnormal liver function tests, neuropathy, decreased bone density and increased risk of fractures, and silent disease (asymptomatic) (6). Initially, CD was considered an ailment of Western society, but data from India, the Middle East and North Africa show prevalence ranging between 0.14 and 1.17% in low risk groups and 2.4 to 4.4% in high risk groups. Most probably this difference is attributable to the heterogeneity of the studied populations, how the subjects were selected, which diagnostic strategies were used, and whether confirmatory biopsies were performed or not (5). The association of ethnicity with the prevalence and pathogenesis of CD has been already studied in England, the United States, Sweden, and Israel (7–10). A recent publication, based on comparisons between Western (Dutch, European, Indonesian, American, Oceanian) and non-Western (Turkish, Moroccan, Cape Verdean, Antillean, Surinamese) ethnicities, demonstrated a significant higher prevalence of CD autoimmunity among 6 years-old children in the Western countries (11).

A report from Israel published in 1984 revealed that the highest incidence rate of CD was found in children born to women of Asian origin and the lowest in second-generation Israeli born mothers (10). Recently, Assa et al. reported that the prevalence of CD in Israel was significantly lower in subjects from lower socioeconomic status backgrounds, as well as those of African, Asian and former Soviet Union origin (12). The presence of CD in Arab children has been the subject of only a few reports and were based only on small case base series (13, 14). The Bedouins in Israel are a subgroup within the minority Arab population, have unique historical, social and cultural characteristics, and an extremely high rate of consanguineous marriage (5, 15). In southern Israel (the Negev desert area), the estimated Bedouin population in 2016 was 246,000 (16). Their lifestyle has changed over the past four decades from semi-nomadic to more urban and less tribal. It is well-known that CD is a polygenic disease. The association between environmental, nutritional habits, genetic (specific haplotype), and ethnicity is still not fully clear. Therefore, we aimed in this study to investigate whether Bedouins have a unique clinical profile (phenotype) at time of diagnosis compared to the Jewish children living in the same geographic area. Secondarily, we aimed to explore whether there is any association between the histological severity of CD and ethnicity.

## Materials and Methods

### Study Population

This retrospective study included patients who were diagnosed between 1997 and 2015 with a positive celiac serology and who underwent gastrointestinal endoscopy and biopsies that confirmed the diagnosis of CD, based on the revised ESPGHAN criteria from 1990 (17). Data were retrieved from the medical records of the pediatric gastroenterology unit at Soroka University Medical Center (SUMC), a tertiary, 1200-bed hospital, located in southern Israel (The Negev desert). SUMC is the only medical center providing primary care facilities for >700,000 residents, almost 35% of whom are Bedouins. Data extracted included: demographics, clinical presentation at diagnosis (intestinal and extra-intestinal manifestation), familial occurrence of CD, additional autoimmune diseases, laboratory results (serology of CD, liver enzymes, hemoglobin levels, iron, ferritin).

### Clinical Data

The clinical picture was classified as intestinal-type CD (typical) if the chief complains were abdominal pain, vomiting, diarrhea or constipation (each one or in combination). Extra-intestinal CD (atypical) was considered if presenting symptoms included iron deficiency anemia (IDA), failure to thrive (FTT), weight loss, short stature, abnormal liver enzymes, presence of other autoimmune diseases, or asymptomatic disease (each one or in combination).

### Age Groups

The cohort was divided to three age groups: Group 1, younger than 3 years old since this is the early presentation of malabsorptive symptoms; Group 2, ages 3–9 because the screening of Bedouin children regarding growth (height and weight) usually takes place before or during second grade; Group 3, 9–18 years.

### Histology

The biopsies were taken according to the ESPGHAN guidelines (6). Those before 2012 included at least four biopsies from the second part of the duodenum, and those since 2012 by at least two biopsies from duodenal bulb and at least four biopsies from the second or third part of the duodenal. Immunohistochemical CD3 staining was performed according to the pathologist discretion, usually in type 2 Marsh criteria.

We used the modified Marsh-Oberhuber classification which categorized as follow: Type 1: Infiltrative lesion, Intra-epithelial lymphocytes (IEL) with normal mucosal architecture. Type 2: Crypt hyperplasia without villous atrophy. Type 3: Villous effacement and crypt hyperplasia. Further categorized as 3a, 3b, and 3c characterized by mild villous atrophy, marked villous atrophy and completely villous atrophy, respectively (18). The grading system proposed by Corazza and Villanacci was considered but not included since the vast majority of the pathologist and clinicians adhere to the Marsh-Oberhuber classification (19).

The study was approved by the local ethics Institutional Review Board of SUMC. The study protocol conforms to the ethical guidelines of the 1975 Declaration of Helsinki as reflected in a priori approval by the institution's Human Research Committee.

### Statistical Analysis

All statistical analyses were performed using SPSS software (IBM SPSS, statistics, Armonk New York, USA), by the statistic department, Tel Aviv University. Mann-Whitney *U*-test or *t*-Test was used in the analysis of ordinal or continuous variables. Chi-square test was used to analyze categorical variables and Fischer's exact test was used where the conditions for using Chi –square test were unmet. A value of *P* < 0.05 was considered statistically significant. Forward stepwise logistic regression analysis was done using Omnibus tests of Model Coefficients.

## Results

### Demographic and Clinical Data

This study included 844 children (age 0–18 years): 505 (59.8%) of them were females, 573 Bedouins (67.9%), and 271 Jewish (32.1%). The mean age at diagnosis was 7.79 (IQR 4.41–10.86) years. Patients were divided according to age at diagnosis into three different age groups; <3 years (Group 1), 3–9 years (Group 2), and >9–18 years (Group 3) (Table 1). The mean age at diagnosis was significantly different between Jewish and Bedouins patients only in Group 2 (age 3–9 yrs.). About half of the patients were between 3 and 9 years old at the time of diagnosis (Table 1).

**Table 1:** A comparison of demographic characteristics between Jewish and Bedouin children.

**Variable**	**Jewish**	**Bedouins**	**Total**	***p*-value**
	***n* = 271**	***n* = 573**	***n* = 844**	
Age Mean	7.63	7.87	7.79	0.455
(IQR) years	(3.85–10.87)	(4.60–10.89)	(4.41–10.86)	
**Age groups, No (%):**				
0–3 years	38 (14.0)	81 (14.1)	119 (14.1)	0.248
3–9 years	139 (51.2)	283 (49.3)	422 (50)	0.049
9–18 years	94 (34.8)	209 (36.6)	303 (35.9)	0.532
**Gender, No (%):**				0.037
Male	95 (35.1)	244 (42.6)	339 (40.2)	
Female	176 (64.9)	329 (57.4)	505 (59.8)	

### Clinical Data of Patients

A third (30.5%) of the Bedouin children with CD presented with typical gastrointestinal manifestations compared to 49.8% of the Jewish children (*p* < 0.01). Bedouins presented more frequently with FTT (24.3 vs. 12.5%, *p* < 0.001), and anemia (62.1 vs. 38%, *p* < 0.001) (Table 2). On the contrary, autoimmune diseases were significantly less frequent in Bedouins at time of diagnosis (*p* = 0.005). A family history of CD was significantly more common among the Bedouins (28.3 vs. 14%) than among the Jewish patients, (*p* < 0.001) (Table 2).

**Table 2:** Clinical data at diagnosis among the different ethnic groups.

		**Jewish**,	**Bedouins**,	**Total**,	***p-*value**
		**No (%)**	**No (%)**	**No (%)**
GI Manifestations	Abdominal pain	97 (35.8)	129 (22.5)	226 (26.8)	<0.01
	Constipation	10 (3.7)	6 (1)	16 (1.9)	0.01
	Diarrhea	43 (15.9)	73 (12.7)	116 (13.7)	0.21
	Vomiting	16 (5.9)	9 (1.6)	25 (3)	<0.01
	All GI Manifestations	135 (49.8)	175 (30.5)	459 (54.4)	<0.01
Extra-GI	FT	34 (12.5)	139 (24.3)	173 (205)	<0.01
Manifestations	Weight loss	14 (5.2)	17 (3)	31 (3.7)	0.11
	Fatigue	12 (4.4)	7 (1.2)	19 (2.3)	0.01
	Short stature	45 (16.6)	117 (20.4)	162 (19.2)	0.19
	Anemia	103 (38)	356 (62.1)	459 (54.4)	<0.01
	IgA deficiency	27 (10)	40 (7)	67 (7.9)	0.12
	Other autoimmune^*^	20 (7.4)	23 (4)	43 (5.1)	0.01
	Diabetes mellitus	15 (5.5)	12 (2.1)	27 (3.2)	0.01
	All Extra-GI Manifestations	155 (57.2)	447 (74.3)	602 (71.3)	<0.01
	Family history	38 (14)	162 (28.3)	200 (23.8)	<0.01

*FTT, failure to thrive; GI, gastrointestinal*.

* *Thyroid, liver, other*.

When divided according to age groups (Table 3), typical gastrointestinal manifestations were also significantly more common in Jewish children, while anemia was significantly more common among Bedouins in all age groups.

**Table 3:** Clinical and histological finding by ethnicity and age groups.

**Age group**	**Ethnic group**	**Typical clinical**	**Anemia, No (%)**	**Subtotal villous**	**Total villous**
		**presentation, No (%)**		**atrophy, No (%)**	**atrophy, No (%)**
0–3 years	Jewish	26 (68.4)	16 (42.1)	23 (60.5)	15 (39.5)
	Bedouins	36 (44.4)	65 (80.2)	39 (48.1)	42 (51.9)
	*p*-value	<0.01	<0.01	0.62	0.21
>3–9 years	Jewish	62 (44.6)	60 (43.2)	99 (71.2)	40 (28.8)
	Bedouins	83 (29.3)	191 (67.5)	158 (55.8)	125 (44.2)
	*p*-value	0.01	<0.01	0.01	0.01
>9–18 years	Jewish	47 (50.0)	27 (28.7)	62 (66.0)	32 (34.0)
	Bedouins	56 (26.8)	100 (47.8)	114 (54.5)	95 (45.5)
	*p*-value	<0.01	0.01	0.05	0.06

### Histological Severity

**Figure 1:**
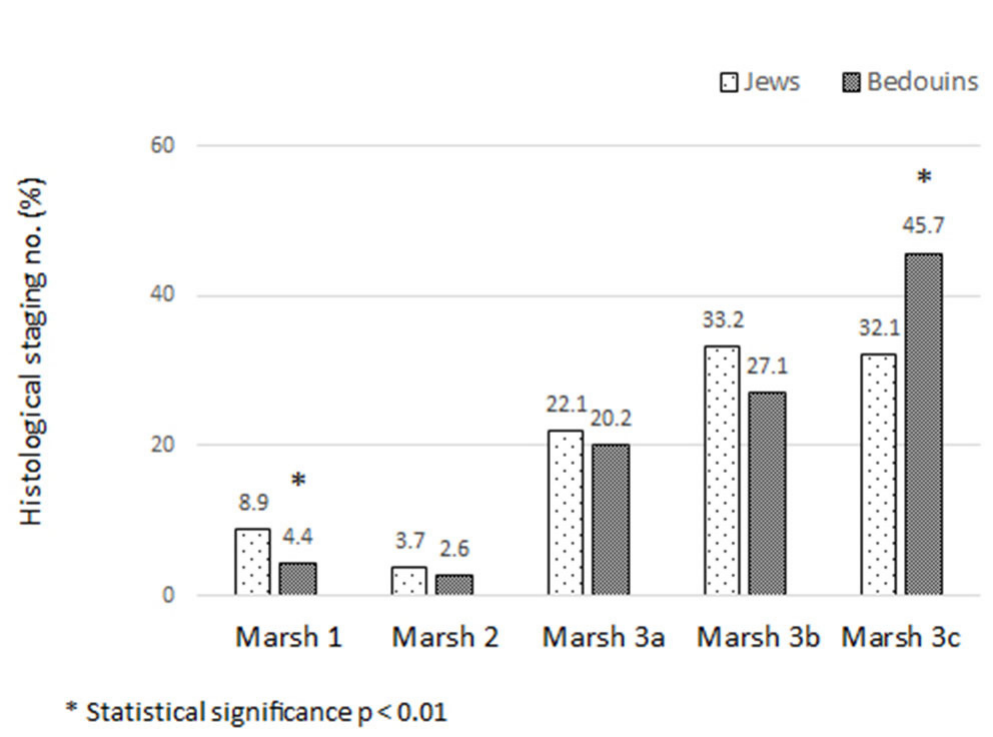
Histological severity among the 2 groups.

The histological severity of CD was determined using the Marsh classification and is presented in Figure 1. Marsh 1 was observed more in Jewish children (8.9 vs. 4.4% in Bedouin, *p* < 0.01) and Marsh 3c more in Bedouins (45.7 vs. 32.1%, *p* < 0.01).

However, analysis by age groups revealed that these differences were statistically significant only in Group 2 (age 3–9 years), (Table 3).

Forward stepwise regression analysis revealed that age at diagnosis was not associated with the histological severity of CD (total villous atrophy-Marsh 3C). On the contrary, Bedouin origin and the presence of anemia at diagnosis were associated with Marsh 3C, with an OR of 2.303 (*P* < 0.009; 95% CI, 1.23–4.30) and an OR of 1.789 (*p* < 0.003; 95% CI, 1.214–2.636), respectively (Table 4).

**Table 4:** Regression analysis.

**All age**	**OR for MARSH**	***p*-value**	**0–3 years**	**OR for MARSH**	***p*-value**
**groups**	**3C (95%CI)**		**Age group**	**3C (95%CI)**	
Bedouins	1.727	0.01	Bedouins	2.02	0.12
	(1.725–2.364)			(0.842-4.483)	
Female	0.9	0.67	Female	0.607	0.21
	(0.706–1.250)			(0.280–1.316)	
Age	1.013	0.43	Age	1.613	0.14
	(0.981–1.047)			(0.856–3.042)	
Typical presentation	1.118	0.46	Typical presentation	1.661	0.2
	(0.830–1.507)			(0.759–3.636)	
Anemia	1.665	0.01	Anemia	0.855	0.74
	(1.241–2.233)			(0.347–2.111)	
**>3–9 years**			**>9–18**		
**age group**			**Age group**		
Bedouins	1.803	0.01	Bedouins	1.507	0.13
	(1.147–2.836)			(0.887–2.561)	
Female	1.041	0.85	Female	0.933	0.78
	(0.692–1.565)			(0.573–1.519)	
Age	1.103	0.12	Age	1.048	0.33
	(0.976–1.248)			(0.955–1.149)	
Typical presentation	1.153	0.52	Typical presentation	0.867	0.59
	(0.747–1.778)			(0.520–1.448)	
Anemia	1.637	0.02	Anemia	2.158	0.01
	(1.066–2.513)			(1.329–3.503)	

## Discussion

Our study indicates that Bedouin children with CD in southern Israel were more likely to have an atypical presentation at diagnosis, especially anemia and the presence of other autoimmune diseases, with less typical gastrointestinal symptoms (abdominal pain, diarrhea, vomiting, and constipation). This unique ethnic group also had more advanced histological CD at time of diagnosis.

The prevalence of CD among the urban Bedouin population in southern Israel was reported to be lower both in pediatric and adult populations (0.51 and 0.12%, respectively) compared to other communities, with a prevalence of about 1% (20).

Previous studies demonstrated that the prevalence of CD differs according to ethnicity: 0.4% in South America, 0.5% in Africa and North America, 0.6% in Asia, 0.8% in Europe and Oceania (21), and in the United states 1% among whites, which was significantly higher than among blacks (0.2%) and Hispanics (0.3%). The incidence of CD in Punjabis (Sikhs, Hindus, and Muslims) is eight times greater than in Gujaratis (Hindus and Muslims) and four times higher than in Leicester, England (22). Data in the Arabic population, especially in Bedouins, is limited, with a prevalence of 0.02% in the Gaza strip (14) and incidence of 1:2800 live births in Jordanian children (13). Additionally, in Sweden there was a decreased incidence of CD in second-generation immigrants and some groups of foreign adoptees, which supports the claim that ethnic genetic heterogeneity may contribute to the variation in CD incidence (9).

The difference in clinical presentation of CD among distinct ethnic groups has not been fully investigated. Published data from England of adult patients with CD showed that Caucasians were older at diagnosis compared to south Asians, presented less frequently with symptoms mimicking “irritable bowel syndrome,” but had more vitamin D and iron deficiency (7). In a Canadian study, south Asian children with CD had more frequent growth concerns at presentation compared to the Caucasian patients. However, the latter group's serologies normalized earlier (23). Comparison between the clinical presentation of Turkish and US patients also supports an association between ethnicity and clinical presentation as diarrhea and anemia were more frequent among Turkish CD children, whereas fatigue, abdominal pain, and bloating were more frequent among American children (24).

Although Bedouins are a unique ethnic group, the HLA DQA1 DQB1 high-risk genotypes associated with CD were found to be similar to those that were observed in northern and southern Europeans (25). Despite the genetic similarity, in our study, the Bedouins presented more with atypical symptoms (anemia, FTT, and fatigue) compared to Jewish children, compatible with a previously published study from Israel (20). These results were unrelated to age at time of diagnosis.

Interestingly, familial occurrence rate of CD was higher among Bedouins than Jewish. This can be explained by the high rate of consanguinity among this population, also manifested by the higher infant mortality rate due to hereditary diseases (26).

There has been limited investigation regarding the impact of ethnicity on the severity of villous atrophy. In China, it was noted that more severe intestinal atrophy and intraepithelial lymphocytosis were seen in Asian patients compared to Caucasians (27). In the United States, the ethnic group with the highest prevalence of villous atrophy compared to other Americans consists of individuals from the Punjab region of India (28). Regarding this topic, there is no published data investigating the histological severity of CD at the time of diagnosis comparing Bedouin children to Jews. In our study total villous atrophy was significantly more common in the Bedouins compared to Jewish children. This significance was confined only to the middle age group (age 3–9 years). It was not seen in the youngest age group (age <3) and had a non-statistically significant trend toward more severity in the group above the age of 9 years old.

The explanation for a more advanced histological disease at the time of diagnosis in Bedouins is probably due to the fact that this unique subpopulation of Arabs suffers from childhood poverty, improper nutrition, and lack of education. This difference was not evident in early childhood perhaps because of breastfeeding in the early years. Furthermore, cultural factors and beliefs may lead to a refusal of diagnostic tests and screening tests of relatives, thus causing a delay in diagnosis. On top of this, perhaps poor compliance with preventive medical services may add to the lack of access to them. This explanation is also supported by an original study of inflammatory bowel disease (IBD) among Bedouins in southern Israel which showed that urbanization affected about 60% of this population and was accompanied by lifestyle changes which led to an increased prevalence rate of IBD (29).

To the best of our knowledge, this study is the first that investigated the clinical presentation, as well as the histological severity, of CD in this unique population and compared it to the Jewish pediatric population in the south of Israel.

Our study has some limitations. It is a retrospective study lacking data regarding compliance, nutritional habits, beliefs, and anthropometric data such as weight, height, BMI, and growth rate.

## Conclusions

Celiac disease is presenting different clinical and histological characteristics depending on ethnicity in the same region of Israel: Bedouins are with more frequent anemia and histological severity, whereas Jewish children are more frequent with gastrointestinal symptoms. It is unclear why this is so, although these differences may be explained either by a delay in diagnosis of the disease, or by certain environmental, cultural, or nutritional factors unique to this ethnic group. Further research within the Bedouin celiac population, including studies to evaluate features of atypical or silent CD, as well as specific genetic markers that are part of the so-called “celiac iceberg,” is certainly warranted.

## Data Availability Statement

All datasets generated for this study are included in the article/supplementary material.

## Ethics Statement

The studies involving human participants were reviewed and approved by Soroka University Medical Center Ethics Committee. Written informed consent from the participants' legal guardian/next of kin was not required to participate in this study in accordance with the national legislation and the institutional requirements.

## Informed Consent

This study design was retrospective and all data were collected anonymously. Therefore, no informed consent was requested.

## Author Contributions

All authors listed have made a substantial, direct and intellectual contribution to the work, and approved it for publication.

## Conflict of Interest

The authors declare that the research was conducted in the absence of any commercial or financial relationships that could be construed as a potential conflict of interest.
